# Atomic insight to lattice distortions caused by carrier self-trapping in oxide materials

**DOI:** 10.1038/srep36929

**Published:** 2016-11-14

**Authors:** Felix Freytag, Gábor Corradi, Mirco Imlau

**Affiliations:** 1 Osnabrueck University, School of Physics, Barbarastraße 7, 49076 Osnabrueck, Germany; 2 Wigner Research Centre for Physics, Institute for Solid State Physics and Optics, Hungarian Academy of Sciences, Konkoly-Thege u. 29-33, 1121 Budapest, Hungary

## Abstract

We gain hitherto missing access to the spatio-temporal evolution of lattice distortions caused by carrier self-trapping in the class of oxide materials - and beyond. The joint experimental/theoretical tool introduced combines femtosecond mid-infrared probe spectroscopy with potential landscape modeling and is based on the original approach that the vibration mode of a biatomic molecule is capable to probe strongly localized, short-lived lattice distortions in its neighborhood. Optically generated, small, strong-coupling polarons in lithium niobate, mediated by OH^−^ ions present as ubiquitous impurities, serve as a prominent example. Polaron trapping is found to result in an experimentally determined redshift of the OH^−^ stretching mode amounting to Δν_vib_ = −3 cm^−1^, that is successfully modeled by a static Morse potential modified by Coulomb potential changes due to the displacements of the surrounding ions and the trapped charge carrier. The evolution of the trapping process can also be highlighted by monitoring the dynamics of the vibrational shift making the method an important tool for studying various systems and applications.

Self-trapping by electron- or hole-phonon-coupling, commonly described as small polaron formation^1^, is increasingly considered in the modeling of transport phenomena in oxide materials such as superconductivity in cuprates^2^, semiconduction in transition metal oxides^3^, colossal magnetoresistance in manganites^4^,^5^, and also for the mechanism of photocatalysis in TiO_2_^6^. In this connection, a detailed knowledge on the lattice distortions related to self-trapping, and in particular on the respective dynamics at elevated temperatures, are of major importance; however, no adequate experimental tools have been described in the literature, so far. Only recently, first attempts for a direct view on small polarons on the surface of TiO_2_ have been reported by Setvin *et al*. based on a combination of scanning tunneling microscopy/spectroscopy and density functional theory^7^. In turn, Sezen *et al*. applied infrared reflection-absorption spectroscopy at low temperatures to study photoexcited polarons in zinc oxide (ZnO)^8^. By contrast, in this article, we intend to access optically generated, small polarons in the crystal bulk at room temperature and propose a time-resolved vibrational method to experimentally achieve local information on small polarons at this temperature.

Our study is performed with nominally undoped, near stoichiometric lithium niobate (LiNbO_3_, LN) as a prototypical oxide material that allows for the optical generation of various kinds of short-lived small, strong-coupling electron and hole polarons^9^, i.e., self-trapped carriers with associated distortions strongly confined to a single lattice site^1^. A multitude of knowledge based on a variety of experimental techniques has been gathered to access small polarons in LN in the past decades. Structural (steady-state) parameters are obtained using a combination of X-ray diffraction/Electron Paramagnetic Resonance (EPR)/Electron Nuclear Double Resonance (ENDOR)/Angle-resolved Photoemission Spectroscopy (APRES) measurements^10^,^11^,^12^,^13^,^14^, which, however, are limited to low temperatures and systems having appropriate spin. Information on the electronic structure is best modeled by ab initio techniques^15^, or, from the experimental viewpoint, using optical pump-probe techniques in the visible spectral range^9^. Access to the dynamics of lattice distortions due to the self-trapping of optically excited carriers, is of importance in LN to foster the understanding of the bulk photovoltaic effect and its small polaron based microscopic modeling^16^.

Our original approach makes use of a molecular ligand in direct vicinity of the small polaron site such that its vibrational spectroscopic fingerprint *E*_vib_ = *hν*_vib_ changes by Δ*ν*_vib_ due to a modified electrostatic potential in the presence of a short-range lattice displacement. With knowledge on the lattice position of both the molecular ligand and the small polaron site, the structural distortion can be reconstructed from the measurement of Δ*ν*_vib_. Thus, a variety of small polaron features like the associated displacements of atomic equilibrium positions or the electron-phonon coupling strength can be obtained. A particular strength of the approach is the possibility to study the temporal evolution of localized atomic displacements if dynamic measurements for the detection of Δ*ν*_vib_(*t*) are applied. Here we use a femtosecond visible pump, mid-infrared (MIR) probe absorption spectrometer (cf. e.g. ref. 17) with a remarkable shot-to-shot sensitivity below 10^−3^ OD (Optical Density) at a spectral and temporal resolution of *δν*_vib_(*t*)/*ν*_vib_ ≈ 9 ⋅ 10^−4^, and *δt* = 1 ms, respectively. It is shown that the transient absorption spectrum Δ*A*(*ν, t*) = *A*(*ν, t*)_pumped_ − *A*(*ν, t*) reveals a frequency shift of Δ*ν*_vib_(OH^−^) upon light exposure that can be related to the formation of short-lived, small O^−^ hole polarons. Furthermore, the dynamic features from ms to seconds in the dark resemble the well-known stretched-exponential behavior that is characteristic for hopping-transport^18^. The main purpose of this article is (i) to verify the underlying experimental approach, i.e., to demonstrate and explain the small polaron access using changes in a vibrational fingerprint, (ii) to sketch the physical relation between experimentally determined frequency shifts and the structural properties of small, strong-coupling polarons, and (iii) to show-up the possibility for dynamic measurements with small polaron hopping-transport as an example.

## Fundamentals

### Carrier self trapping in lithium niobate

**Figure 1:**
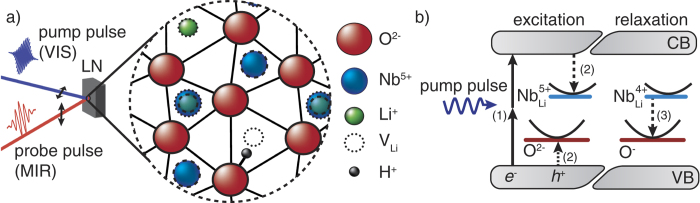
(**a**) Structure of the oxygen plane of LiNbO_3_^10^ (dotted: cations above plane, dashed: below plane) and schematic setup. (**b**) Electronic transition: (1) two-photon-absorption, (2) generation of small 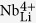 electron and O^−^ hole polarons (*τ* < 1 ps)^41^, (3) recombination (*τ* ≈ 1 s) (Table 1).

In what follows, we will focus on small, strong-coupling O^−^ hole polarons (HP)^19^ in direct vicinity to a V_Li_ vacancy as well as on 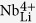 electron polarons (GP)^20^. Structural details of the oxygen plane of LN are depicted in Fig. 1a. In both cases the trapped charge can be transferred by light to a neighboring equivalent or nearly equivalent site, thereby stripping the trapped charge of its polarization halo^18^,^19^. This results in broad absorption features playing a central role in the study and application of dynamic properties of small polarons in LN^9^. The absorption maxima are reported in the visible region (VIS, 2.5 eV; ≈20,000 cm^−1^) for HP and in the near infrared (NIR, 1.6 eV; ≈13,000 cm^−1^) for GP. In nominally undoped LN, pairs of HP and GP can be generated via two-photon excitation at 2.5 eV (cf. Fig. 1b, left part), i.e., with photon energies much below the band edge energy (E_gap_ ≈ 4.1 eV^21^). To raise signal changes in the MIR range (≈10 mOD, see below), it is necessary to generate a large number density N_sp_ > 10^18^ cm^−3^ of small polarons. This is realized by using intense laser pulses (*I*_pump_ = (500 ± 100) TW/m^2^, extraordinary light polarization) with sub-ps pulse duration (*τ*_pump_ = (120 ± 10) fs), as *N*_sp_ depends quadratically on the intensity of the pump-pulse, 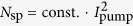
22, but also on the pulse duration *τ*_pump_^23^. Furthermore, repetitive fs-pulse exposure (*f*_rep_ = 1 kHz) is applied, which leads, after a few seconds of illumination, to a saturation of the MIR effect. The average small polaron lifetimes *τ*_GP_ ≈ *τ*_HP_ ≈ (1.1 ± 0.3)s ≫ 1/*f*_rep_ accord with the mutual HP-GP-recombination path, as shown in Fig. 1b, right part.

### Hydroxide ions as a molecular probe

**Figure 2:**
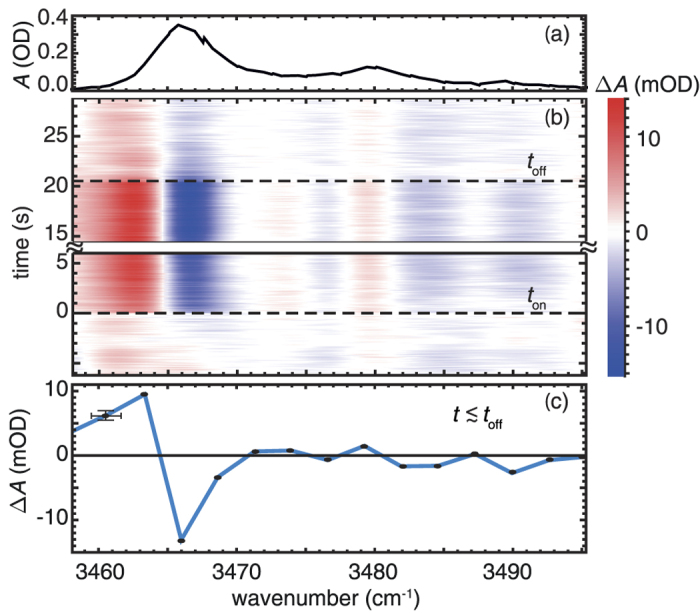
(**a**) Steady state spectrum of the OH^−^-stretching absorption feature in nearly stoichiometric lithium niobate. (**b**) Time dependent change of the absorption 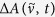 of the OH^−^- stretching vibration. *t*_on_ and *t*_off_ denote the times where repetitive pump-pulse exposure is switched on and off. (**c**) Spectrum of the absorption change at *t* = *t*_off_ (black data points). The blue line is a guide to the eye; its shape indicates a frequency shift of the prominent absorption feature by −3 cm^−1^.

Hydroxide ions, OH^−^ 13, inevitably introduced during crystal growth are used as molecular probes. Real position and vibrational properties have been studied in LN crystals comprehensively (for reviews, cf. refs 24 and 25), showing that H^+^ is bound to one of the O^2−^ neighbors of a V_Li_^26^,^27^,^28^ (note that 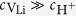
26). Due to the dipolar character of this defect complex it may be assumed that light-induced, small polarons are formed or get temporarily trapped in its direct vicinity. We consider an O^−^ hole polaron localized at an oxygen ion next to the same V_Li_ (see Fig. 1a). It is already established that the energy of the OH^−^-stretching vibration depends on the surroundings of the particular OH^−^-site resulting in several components of its MIR absorption feature. In as-grown, near stoichiometric LN up to five distinct bands have been observed^29^,^30^; the most prominent bands appear at 3,466 cm^−1^, 3,480 cm^−1^, and 3,490 cm^−1^ (see Fig. 2a) with the lowest vibrational energy related to the O^2−^ sites closest to V_Li_^26^. To observe pump-induced frequency changes of the OH^−^-stretching bond, we use probe pulses with a spectral width of 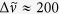 cm^−1^ at a center wavenumber of 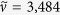 cm^−1^ (*I* = 0.03 TW/m^2^, *τ*_probe_ = (230 ± 20) fs, *f*_rep_ = 1 kHz, ordinary light polarization). All studies were performed with an X-cut sample (aperture 10 × 9 mm^2^, thickness 2 mm) with a [Li]/[Nb] ratio of 0.992^31^ and a hydrogen concentration^32^ of ≈ 3 ⋅ 10^18^ cm^−3^.

## Results

### Experiment

The time dependent change of the absorption feature 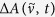 in the region of *ν*_vib_(OH^−^) is plotted as a function of wavenumber 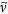 in Fig. 2b using the color coding: red for 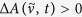 and blue for 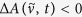. In the time regime *t* < *t*_on_, i.e., without the presence of the pump-pulse, we detect a weak background signal that is applied for signal correction. At *t*_on_, the repetitive pump-pulse exposure is switched on and induces spectral changes 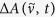 well above the noise limit. The temporal evolution resembles a mono-exponential behavior with a characteristic time constant in the order of 100 ms. The spectral evolution shows changes in the shape of all three MIR absorption bands with maximum values of about −14 mOD at 3,466 cm^−1^ and about +10 mOD at 3,463 cm^−1^. Pump-induced changes at 3,480 cm^−1^ and 3,490 cm^−1^ are also observed but do not exceed 2 mOD; accordingly we will focus in the following on the strongest absorption band. Switching off the pump-pulse exposure (*t* > *t*_off_), the absorption change decays to nearly zero and the process can be repeated from the beginning. Exemplarily, the spectrum 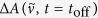 is extracted and plotted in Fig. 2c. The remarkable shape, in particular in the region of the strongest absorption band, can be understood as a shift of the OH^−^ absorption frequency by −3 cm^−1^ to 3,463 cm^−1^.

**Figure 3:**
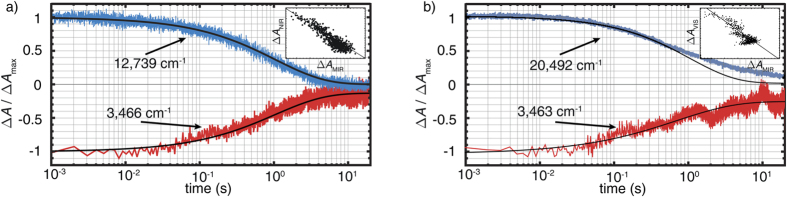
Relaxation dynamics probed at (**a**) 3,466 cm^−1^ (MIR) and 12,739 cm^−1^ (*λ* = 785 nm, NIR) and (**b**) at 3,463 cm^−1^ (MIR, inverted) and 20,492 cm^−1^ (*λ* = 488 nm, VIS). The solid lines indicate the results of fitting stretched exponential functions to the data sets. Insert: Plot of the absorbance change (**a**) Δ*A*_NIR_, (**b**) Δ*A*_VIS_ as a function of Δ*A*_MIR_; a linear dependence is revealed over the entire data range.

The next step is to verify the relation of this shift to the presence of small, strong-coupling polarons. For this purpose, the temporal relaxation dynamics in the mid-infrared spectral range is highlighted in a semi-logarithmic plot in Fig. 3 (red data points) and compared with the temporal decay dynamics of the light-induced absorption detected in the near-infrared and visible spectral range (blue data points) close to the maximum of the GP and HP absorption features. It is evident from Fig. 3 that the temporal behaviors of the absorption changes in all spectral ranges are strongly correlated, despite the differences in the sign of Δ*A*. As shown in the inserts of Fig. 3, the Δ*A*_NIR/VIS_(Δ*A*_MIR_) plot is essentially linear. For a quantitative comparison, a stretched-exponential function


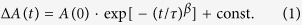


with time constant *τ* and stretching exponent *β* is fitted to each data set. The values (*τ*,*β*) obtained from fitting the experimental data are listed in Table 1 and are comparable within the error margins. These findings are particularly remarkable since the absorption in the NIR and VIS region is related to changes in the electronic structure, whereas the absorption in the MIR is characteristic for the molecular vibration of OH^−^. The linear relation between the signals of different origin, thus, gives clear evidence for their common physical background which is the hopping transport of GP and HP leading to their recombination (cf. Fig. 1b). We note that the absorption dynamics at 3,463 cm^−1^ in Fig. 3b shows a reduced signal-to-noise ratio in comparison with data at 3,466 cm^−1^, that is due to the lower signal level at this wavenumber. Concerning the dynamics at 20,492 cm^−1^, a slight variation to long decay times in comparison to the stretched-exponential decay is observed, that may be attributed to the presence of 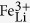 impurities, always present in the ppm regime in nominally undoped lithium niobate and acting as deep electron traps. At the same time, the frequency shift cannot be explained by other defect centers such as extrinsic dopants, as a change in the cation environment, caused, e.g., by doping, leads to a blue shift of the MIR absorption spectrum (e.g. magnesium, indium or neodymium shift the frequency by a few +10 cm^−1^ 25,33). Electron polarons bound to 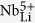 antisites can be discarded in this respect as protons are not expected to associate with antisites due to the huge surplus positive charge of the latter. Small, free polarons on a nearby regular 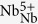-site would have much shorter relaxation times (*τ* ≈ 1*μ*s)^34^ and can also be excluded.

### Theory

**Figure 4:**
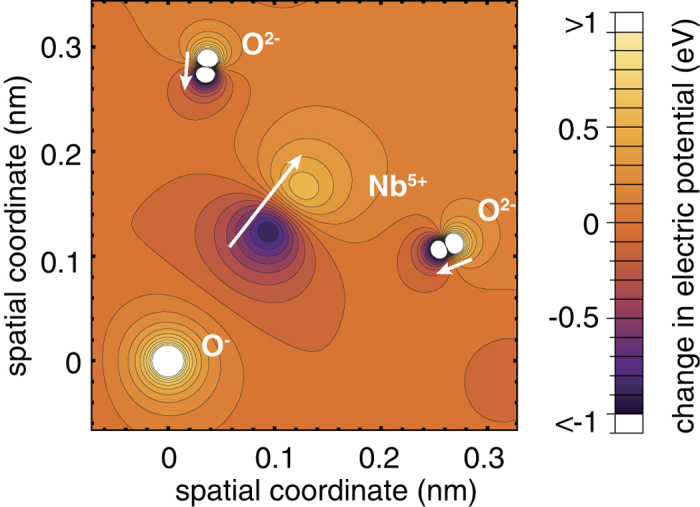
Electrostatic potential change in the oxygen plane of LiNbO_3_ calculated for a polaronic distortion corresponding to a redshift of −3 cm^−1^ of the OH^−^-stretching vibration. Arrows (enlarged) indicate amplitudes and directions of displacements (projected to the oxygen plane) of close Nb^5+^ and O^−^ neighbors of the polaron host that is positioned in the origin. Note that the Nb^5+^ site is ≈ 0.088 nm below the oxygen plane.

In the final part of this letter, based on the experimental findings, we give an estimate of the local lattice distortion induced by small hole polarons, using a simple point charge approximation for the change of the crystal field and a static Morse potential^35^ along the OH^−^-bond with the anharmonicity constant *ω*_*e*_*x*_*e*_:


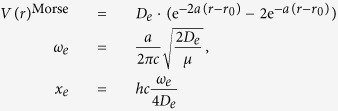


with *D*_*e*_ being the dissociation energy, *a* the stretching constant, *r*_0_ the O-H distance with the lowest potential energy and 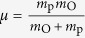 the reduced proton mass. In the absence of polarons the values of *D*_*e*_ and *a* can be found using measured frequencies (*ν*_0,1_ = *ω*_*e*_ − 2*ω*_*e*_*x*_*e*_ = 3,466 cm^−1^, *ν*_0,2_ = 6,745 cm^−1^) of the OH^−^-stretching vibration^36^. A hole polaron is added at another oxygen neighbor of the lithium vacancy out of the three nearest ones. The potential change seen by the proton is accounted for by the Coulomb potential due to the added point charge on its host ion *and* by the changes of the Coulomb potentials due to the displacements of all neighboring ions with the exception of the oxygen in OH^−^. For simplicity the displacements are assumed to be radial (with respect to the polaron host) and scaled by the factor *q*_i_/*d*_i_ where *q*_i_ is the charge and *d*_i_ the distance (to the host) of the i^th^ neighbor (see Fig. 4). This scaling corresponds to considering only long range electron-phonon interaction characterized by the static dielectric constant *ε*_0_ having a large value in comparison to the optical dielectric constant *ε*_∞_ (*ε*_0,11_/*ε*_∞_ = 57.6, *ε*_0,33_/*ε*_∞_ = 19.0^37^, with *ε*_∞_ ≈ 1.47 ⋅ *ε*_vacuum_^38^) in LN. Choosing the displacement *δd*_Nb_ of the nearest Nb neighbor (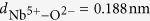) as a numerical scaling parameter and neglecting possible changes of the OH^−^-direction, the resulting potential along the bond can be approximated by a new Morse potential of the same type.

The three dimensional Coulomb potential is calculated for a 5 × 5 × 2 hexagonal unit cell (1588 atoms), using the structural data of the LiNbO_3_ lattice^10^ with a lithium and an adjacent oxygen ion at the center of the cell replaced by an OH^−^-molecular-ion represented by a static Morse potential. The site of the proton substituting V_Li_, is assumed to be in the bisecting plane of the largest oxygen triangle (O-O distance 336 pm)^28^ with an O-H bond length of 98.8 pm and an out-of-plane angle of 4.3 degrees towards V_Li_^27^. We note that a OH^−^ − V_Li_-complex causes only negligible distortions of the lattice as calculated by Lengyel *et al*.^25^,^27^. Cation charges in the boundary area of the cell are reduced to secure an overall neutral charge. The changes of the potential along the direction of the O-H bond resulting from the presence of a hole polaron are added to the Morse potential, neglecting the difference between the center of mass of OH^−^ and the corresponding oxygen site. The hole polaron state is modeled by an additional positive charge localized at one of the oxygen atoms in closest vicinity to the V_Li_-site and by scaled displacements of the surrounding atoms within a radius of 5 Å.

For *δd*_Nb_ = 0.024 nm the modified Morse potential returns the shifted fundamental frequency 3,463 cm^−1^ found by our experiments. Taking into account only the point charge of the polaron without lattice relaxation, a blue shift of +5 cm^−1^ is obtained which stresses the importance of the polaronic relaxation. The obtained ≈12.5% increase of the smallest O-Nb distance corresponds to a ≈1.7% increase of the closest O-Li distance (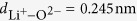), and an average ≈2.1% decrease of the various O-O distances (between 0.272 nm and 0.336 nm), taking into account the adopted *q*_i_/*d*_i_ scaling (see Fig. 4). Considering this scaling our calculations are consistent with recent ab initio studies of the polaronic deformation at the impurity site in Fe:LiNbO_3_ by Sanson *et al*.^39^ who found a ≈4.7% decrease of the Fe-O distance (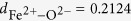 nm) upon hole capture by Fe^2+^, thus, supporting our model assumptions. The estimates derived from our simplified model may already serve as useful input parameters for more complex model calculations and/or as a crosscheck of the validity of various small polaron model approaches. Appropriate calculations may be used straightforwardly for the determination of further small polaron parameters, like the electron phonon coupling strength.

## Summary and Conclusions

Summarizing the results, a pump-induced −3 cm^−1^ shift of the frequency of the OH^−^-stretching vibration is experimentally verified and attributed to the presence of small, strong-coupling O^−^ hole polarons next to the OH^−^ ion. Sign and magnitude of the shift can only be explained by taking into account, in addition to the polaronic charge, also the distortion of the lattice in the imminent neighborhood - a very striking result that supports the microscopic approach for the bulk photovoltaic effect based on small, strong-coupling polarons in LN^16^ and will foster its advances. The measured shift can be reconstructed by modeling the change of the three-dimensional Coulomb potential caused by the polaron, leading to reasonable values of the ligand displacements. The measured frequency shift may also provide a stringent test for ab initio/DFT calculations desirable for a deeper understanding of the properties of polarons interacting with molecular defects, and vice versa. It should be stressed that the presented experimental approach for determining local lattice deformation, although introduced with small, strong-coupling polarons, can also be applied for the detection of small, weak-coupling and large polarons^1^. It is furthermore neither limited to the chosen molecular hydoxide group nor to lithium niobate as a host material, and may be adopted, e.g., to further oxides like TiO_2_, ZnO or MgO, but also to probe localized atomic distortions in DNA^40^. As its most important aspect, however, the approach enables dynamic studies at room temperature and, thus, general physical questions about polaron formation, hopping-transport and recombination phenomena, encompassing time scales from sub-ps to seconds, become accessible. If necessary, the time resolution can be increased to the sub-ps time regime by using up-conversion MIR-spectroscopy; this enables a larger signal-to-noise ratio accompanied with a better time resolution.

## Additional Information

**How to cite this article**: Freytag, F. *et al*. Atomic insight to lattice distortions caused by carrier self-trapping in oxide materials. *Sci. Rep.* **6**, 36929; doi: 10.1038/srep36929 (2016).

**Publisher’s note**: Springer Nature remains neutral with regard to jurisdictional claims in published maps and institutional affiliations.

## Figures and Tables

**Table 1:** Results of fitting a stretched-exponential function to the temporal dynamics in the VIS, NIR and MIR spectral range (cf. straight lines in Fig. 3).

Probed effect	Range	*ν*_probe_/cm^−1^	*τ*/s	*β*
Molecular vibration	MIR	3,463	0.7 (4)	0.55 (10)
		3,466	1.0 (4)	0.65 (10)
Electronic transition	NIR	12,739	1.1 (3)	0.65 (10)
	VIS	20,492	1.1 (3)	0.70 (10)
